# VEGF-A and FGF4 Engineered C2C12 Myoblasts and Angiogenesis in the Chick Chorioallantoic Membrane

**DOI:** 10.3390/biomedicines10081781

**Published:** 2022-07-23

**Authors:** Donna C. Kennedy, Antony M. Wheatley, Karl J. A. McCullagh

**Affiliations:** Department of Physiology, School of Medicine, Human Biology Building, University of Galway, H91 W5P7 Galway, Ireland; d.kennedy12@nuigalway.ie (D.C.K.); antony.wheatley@nuigalway.ie (A.M.W.)

**Keywords:** skeletal muscle, angiogenic, vascular endothelial growth factor-A, fibroblast growth factor, endothelial cells, stable cell line, CAM

## Abstract

Angiogenesis is the formation of new blood vessels from pre-existing vessels. Adequate oxygen transport and waste removal are necessary for tissue homeostasis. Restrictions in blood supply can lead to ischaemia which can contribute to disease pathology. Vascular endothelial growth factor (VEGF) is essential in angiogenesis and myogenesis, making it an ideal candidate for angiogenic and myogenic stimulation in muscle. We established C2C12 mouse myoblast cell lines which stably express elevated levels of (i) human VEGF-A and (ii) dual human FGF4-VEGF-A. Both stably transfected cells secreted increased amounts of human VEGF-A compared to non-transfected cells, with the latter greater than the former. In vitro, conditioned media from engineered cells resulted in a significant increase in endothelial cell proliferation, migration, and tube formation. In vivo, this conditioned media produced a 1.5-fold increase in angiogenesis in the chick chorioallantoic membrane (CAM) assay. Delivery of the engineered myoblasts on Matrigel demonstrated continued biological activity by eliciting an almost 2-fold increase in angiogenic response when applied directly to the CAM assay. These studies qualify the use of genetically modified myoblasts in therapeutic angiogenesis for the treatment of muscle diseases associated with vascular defects.

## 1. Introduction

Adequate oxygen transport and waste removal are essential for tissue homeostasis. For this to occur, a vast network of well-connected blood vessels to deliver well-oxygenated blood is required, with ischaemia occurring in its absence leading to tissue deprivation and death [1,2]. Angiogenesis is the process by which new blood vessels form from pre-existing vessels, and which occurs both physiologically and in malignancy [3]. Alterations in angiogenesis are observed in primary and secondary muscle diseases which can be indicative of a poorer prognosis in conditions such as critical limb ischaemia (CLI), peripheral artery disease (PAD), diabetes, cachexia, chronic obstructive pulmonary disease (COPD) [4,5] and has been proposed as a contributory factor in the pathology and progression of Duchenne muscular dystrophy (DMD) [6].

Therapeutic angiogenesis is the coordinated delivery of vascular growth factors with the aim of promoting blood vessel growth, leading to revascularisation of previously under-perfused tissues [7,8]. Vascular endothelial growth factor (VEGF) is paramount in the induction of angiogenesis, but it may also dually promote myogenesis in vivo [9,10]. In vitro studies have shown the presence of VEGF receptors -1 (VEGFR-1) and -2 (VEGFR-2) on the surface of both C2C12 and primary mouse myoblasts, with the expression of these receptors further increased following cell differentiation [10]. Increased levels of VEGF-A present in myoblast cell cultures have also resulted in increased numbers of multinucleated myosin heavy chain positive cells, increased cell migration, reduced cell proliferation, and reduced levels of apoptotic cells compared to untreated myoblasts [10,11,12].

VEGF-A is a key factor in vascular development and is the single most important regulator of blood vessel formation [13]. VEGF exists in several isoforms, which developed from alternative mRNA splicing [14], VEGF-165 being the predominant isoform, showing a significant potency and consequently, the strongest therapeutic potential [14,15,16]. Working through the coupling to tyrosine kinase receptors VEGFR-1 and VEGFR-2, VEGF has the ability to stimulate the migration and proliferation of the endothelial cells required for the assembly of the new blood vessels [17,18,19]. However, despite its potent angiogenic potential, studies have indicated that the beneficial dosage for VEGF has a narrow window, with overproduction leading to the development of aberrant blood vessels [20]. VEGF-A and fibroblast growth factor (FGF) are the most commonly described angiogenic growth factors [21]. FGF4 has a synergistic effect with VEGF-A, both through autocrine and paracrine functions [22], with VEGF-A an important downstream mediator of the mitogenic activity of FGF [23,24]. FGF regulates several endothelial cellular functions, such as proliferation, survival, migration, and motility, while also maintaining endothelial cell barrier integrity through activation of tight junctions [23]. Therefore, it has been suggested that the co-delivery of VEGF with other growth factors such as fibroblast growth factor (FGF), may counteract this inadequate vascularisation and promote healthier less leaky, mature blood vessels [18,25,26]. 

The C2C12 immortal muscle cell line has a rapid proliferative and differentiation ability making it an ideal in vitro approach to studying myogenesis [27]. VEGF-A has been stably transfected into C2C12 muscle cells, with its expression analysed through molecular analysis, however, the angiogenic effects of these cells have not yet been fully explored in vivo [18,28].

Previous studies have reported the implantation of VEGF-expressing muscle-derived stem cells improves vascularisation and reduces fibrosis in dystrophic muscle [29,30] and improves capillary density in skeletal and cardiac muscle defects [31,32]. With several approaches possible for the delivery of VEGF into muscle tissue, AAV delivery of VEGF directly into muscle has proven efficacious [33]. The delivery of VEGF-producing muscle cells as an ex vivo approach is advantageous, as VEGF has the potential to both induce angiogenesis and trigger the propagation of muscle cells, restoring damage caused by disease. VEGF has been shown to be effective in increasing myoblast differentiation [10,34] and migration [11], while also preventing cell apoptosis [10,11,12]. However, reports on the effect of VEGF on myoblast proliferation are inconsistent [10,12,26]. 

Several angiogenic assays, both in vitro and in vivo have been used in the past to study angiogenesis. These include in vitro assays such as the use of mouse and human endothelial cell lines (migration, proliferation, survival, and morphogenesis assays) [17] and in vivo assays including the mouse dorsal skin fold [35,36] and the chick chorioallantoic membrane (CAM) assay [37]. We have chosen to focus on using the CAM assay and an endothelial cell line to adequately assess the angiogenic potential of the *hVEGF-A* secreting stable cells. The chick CAM is a highly vascularised membrane used for nourishment and gaseous exchange in fertilized chicken eggs [37,38]. With a vast vascular network of capillaries, veins, and arteries, the chick CAM can be easily manipulated and observed for experimental study [39,40]. Matrigel is an extracellular matrix (ECM) gel formed from mouse sarcoma, a liquid when cooled, and polymerizes when warmed to form a stable gel-like scaffold which can be used to encapsulate cells applied to the CAM assay [41,42,43].

We used the CAM and endothelial cell proliferation, migration, and morphogenesis assays to develop an ex-vivo strategy to deliver the angiogenic factor *hVEGF*, both alone and as part of a dual plasmid co-expressing *hFGF4* and *hVEGF*. We successfully characterised the *hVEGF-A* expression and the angiogenic potential of stably transfected C2C12 mouse myoblast cells using these models of angiogenesis. The first aim was to establish a stable C2C12 muscle cell line expressing *hVEGF-A* protein, confirmed through molecular analysis. Secondly, we observed the paracrine angiogenic efficacy of conditioned media taken from these cells using the CAM filter disc and endothelial cell assays. Finally, we investigated the in vivo pro-angiogenic efficacy of these cells when applied to the CAM angiogenic assay. This investigation combines elements of several previous studies, providing an insight into whether *hVEGF-A*-producing cells can be used as a pro-angiogenic factor with therapeutic potential in the treatment of conditions that experience decreased neovascularisation.

## 2. Materials and Methods

### 2.1. Cell Culture

C2C12 (ATCC, #CRL-1772) mouse myoblast cells and C166 (ATCC, #CRL-2581) mouse yolk sac endothelial cells were cultured in high glucose Dulbecco’s Modified Eagle medium with sodium pyruvate, supplemented with 1% penicillin-streptomycin antibiotic and 10% fetal calf serum (Sigma-Aldrich, Wicklow, Ireland). Cells were maintained in the standard cell culture conditions (5% CO2 and 37 °C) and sub-cultured when necessary, using trypsin/EDTA solution.

### 2.2. Establishment of G418 Antibiotic Kill Curve

The primary aim of the study was to establish a stable muscle cell line to express VEGF-A. To this aim, an antibiotic kill curve was established to determine the minimum concentration of antibiotics required to select positively transfected C2C12 cells. Each of the three plasmids used in this study contained a Geneticin (G418) resistance gene. The kill curve experiment involved a dose-response where non-transfected C2C12 cells were seeded at low (0.1 × 10^6^) and high (0.2 × 10^6^) densities and subjected to increasing concentrations (0–1000 μg/mL) of G418 to determine the minimum antibiotic concentration needed to kill all cells over the course of 3- 7 days. Cell confluency was assessed by visual inspection, while cell numbers were counted using the Countess™ II FL Automated Cell Counter (ThermoFisher Scientific) following 7 days of exposure. At 1000 µg/mL of G418, there was a visible reduction in confluency and increased cell toxicity in those seeded at both low and high concentrations (Figure S1A). Above a G418 concentration of 800 µg/mL, less than 10% of cells seeded at both low and high densities survived for the entirety of the experiment. However, a high dose effect of toxicity and reduced viability was apparent earlier at a concentration of 1000 µg/mL, with most cells dead by day 4 (Figure S1B–E). As a result, 1000 µg/mL G418 antibiotic was determined as the most appropriate concentration for the screening of resistant stable transfected C2C12 cells.

### 2.3. Plasmid Preparation

The pVEGF-EGFPN1 and pEGFPN1 plasmids were received from Dr. Tamas Balla at the National Institutes of Health, Bethesda, USA, while pTR-UF-22-FGF4-ires-VEGF (denoted as FGF4/VEGF) was received from Prof Jozef Dulak, Department of Biochemistry, Biophysics, and Medical Biotechnology Jagiellonian University, Krakow, Poland. Heat shock was used to transform plasmids into DH5α competent E. coli cells, and subsequently cultivated. The DNA was then isolated using a Miniprep DNA Elution Kit (Qiagen, Hilden, Germany) according to the standard manufacturer’s instructions. Confirmation of the plasmids was carried out by DNA restriction digests and gel electrophoresis.

### 2.4. Stable Transfection

Transfections with pVEGF-EGFPN1, pEGFPN1, and FGF4/VEGF plasmids were carried out using Lipofectamine 2000 (Invitrogen), in a 1:2.5 ratio of DNA: Lipofectamine, with a DNA concentration of 8 µg for each plasmid. The DNA and Lipofectamine mixture was applied to plates containing C2C12 cells at 90% confluency and incubated at 37 °C overnight. In the case of pVEGF-EGFPN1 and pEGFPN1 plasmids, positive transfection was identified by observing GFP positive cells under FITC light 24 h post-transfection. Plates were trypsinised, serially diluted, and seeded in Geneticin (G418) selective media to screen for positively transfected cell colonies. Plates were screened for antibiotic resistance for four weeks, with media replenished every 3 days until isolated single colonies could be observed. Isolated colonies which showed continued antibiotic resistance were isolated using trypsin and cloning discs and expanded for further study with two clones selected for each pVEGF-EGFPN1 and pEGFPN1 plasmid, and eleven clones selected for FGF4/VEGF.

### 2.5. Human VEGF-ELISA of Conditioned Media

Non-transfected and stable transfected C2C12 cells were seeded and allowed to grow for 72 h. Media taken from each well was then centrifuged at 1000 rpm for 10 min to remove cell debris and frozen in aliquots at −80 °C. Protein levels of VEGF-A were determined using an *hVEGF-A* ELISA kit (Invitrogen) according to the manufacturer’s instructions. Conditioned media from non-transfected C2C12 myoblasts served as a control. Selected cell lines for each plasmid were designated as Myo-GFP, Myo-VEGF-GFP, and Myo-FGF4-VEGF and were each expanded for further investigation into their angiogenic potential.

### 2.6. Gene Expression Analysis Using Quantitative Real-Time PCR (qPCR)

Gene expression of cells was examined at the mRNA level using qPCR analysis and gene-specific primers. RNA was isolated from confluent non-transfected and stable transfected C2C12 cells using TRIzol^®^ Reagent (Invitrogen). The RNA samples were quantified using a NanoDrop ND 1000 spectrophotometer (ThermoFisher Scientific, Waltham, MA, USA). A total of 500 ng total RNA was reverse transcribed into first-strand cDNA using Superscript III Reverse Transcriptase with random primers (ThermoFisher Scientific). qPCR was then performed using a StepOne Plus RealTime PCR system (Applied Biosystems Waltham, Massachusetts, USA). Each 20 μL reaction mixture consisted of 5 μL cDNA, 5 μL SyberGreen, and 10 mmol/L of specific primers (Supplementary Table S1). The relative gene expression levels were normalised using the S29 housekeeping gene, calculated using the ΔΔCt method allowing quantification of upregulation or downregulation of specific genes [44].

### 2.7. Endothelial Cell Migration (Scratch) Assay

Endothelial cell migration (Scratch) assay was carried out as described previously [45,46]. C166 cells (ATCC, #CRL-2581) were seeded in a 12-well plate at a density of 0.2 × 10^6^ cells/well and left overnight. A P200 pipette tip was used to scratch horizontally across each well, followed by a wash with PBS to remove cell debris, and conditioned media from Myo (control) or Myo-GFP, Myo-VEGF-GFP and Myo-FGF4-VEGF cells were applied. Scratches were imaged using EVOS M5000 at 10X magnification and then returned to the incubator at 37 °C for 8 h before being imaged again. Cell migration was quantified by the change in width of the scratch between experimental time points, with the distance measured using Image J software (Version 1.52a, U. S. National Institutes of Health, Bethesda, MD, USA. https://imagej.nih.gov/ij/, accessed on 8 April 2019).

### 2.8. Endothelial Cell Proliferation Assay

Endothelial cell proliferation was measured using a mitochondrial metabolic (MTT) activity assay, similarly to as described previously [47,48]. C166 cells were seeded in 96 well plates (2000 cells/well) and allowed to attach overnight. DMEM was then removed and replaced with fresh DMEM or conditioned media taken from confluent Myo (control), Myo-GFP, Myo-VEGF-GFP, and Myo-FGF4-VEGF cells. After 72 h, media was removed from each well and replaced with 100 μL MTT solution (5 mg/mL). The plate was then incubated at 37 °C for 4 h, the media was carefully removed and 100 μL of a DMSO/Isopropanol mix was added to each well. The plate was then shaken for 10 min, and the absorbance was measured at 590 nm using a Hidex Sense microplate reader (Hidex, Turku, Finland). Data were averaged from measurements of 8–10 replicates of each treatment, with cell proliferation normalised to fresh DMEM.

### 2.9. Endothelial Cell Tube Formation Assay

Endothelial cell tube formation assay was carried out as described previously [49,50]. 96-well plates were prepared through the addition of 50 μL of 10 mg/mL Matrigel (High Concentration Matrigel, Cat. No. 354248, BD Biosciences) per well. C166 cells were seeded at a density of 2.5 × 10^4^ in triplicate in 100 μL of conditioned media taken from Myo (control), Myo-GFP, Myo-VEGF-GFP, or Myo-FGF4-VEGF cells. After 18 h, tube-like structures were observed. Each well was imaged at four non-overlapping locations, and the number of tubes, nodes, and total tube length were measured using Image J software (Figure S2).

### 2.10. Chick Chorioallantoic Membrane (CAM) Assay

The CAM assay was carried out similarly to as described by others [51,52,53,54]. Fertilized white leghorn chicken eggs were obtained from Shannon Vale Foods and St David’s Poultry Team Ireland, Co. Limerick, Ireland and incubated at 10 °C until activation. A total of 40 eggs were used for each experiment. On activation day (EDD1) eggs were transferred to the Ova-easy advanced series II incubator (Brinsea, Weston-super-Mare, UK) until EDD4, rotating eggs every 30 min at a constant temperature of 37 °C and a humidity of 44%. On EDD4, in aseptic conditions, a square window was made on the surface of the eggshell using a handheld rotator tool, the eggs were resealed with a plastic cover and then transferred to a Memmert HPP260 Climate Incubator, with the temperature of 37 °C and humidity of 80% and left undisturbed until EDD8 when the CAM assay took place.

### 2.11. Conditioned Media CAM Filter Disc Assay

The CAM filter disc assay was carried out as previously described with minor modifications [55,56]. Whatman filter paper was cut into 6 mm diameter filter discs using a hole punch and sterilized in a UV box for 1 h. Conditioned media was taken from confluent Myo (control), Myo-GFP, Myo-VEGF-GFP, and Myo-FGF4-VEGF cells. The media was centrifuged at 1000 RPM for 10 min and 20 µL was then applied to the filter disc and allowed to dry. Using sterile forceps, the discs were then applied to the CAM, and an additional 20 μL of media was then applied, to create a double-dose effect. Two on-plants of the same treatment group were applied to each CAM [57,58]. The eggs were then returned to the incubator until EDD11 where they were sacrificed by placing on ice for 30 min in preparation for imaging and analysis. Surgical scissors and forceps were used to gently lift the CAM and cut it from the rest of the egg. The CAM was then rinsed off in PBS, turned over to view the underside of the on-plant, and imaged immediately using the Leica surgical microscope and camera (Leica M651; Leica Mikroskopie &System GmbH, Wetzlar, Germany) [59]. Each experiment was repeated twice, with results combined to ensure enough data points for a reliable statistical analysis [60].

### 2.12. Matrigel-Cell CAM Assay

The Matrigel-cell CAM assay was carried out similarly to previously described with minor modifications [60,61]. Nylon mesh was cut into 4 × 4 mm squares and sterilized in a UV box for 1 h. A Matrigel-cell mixture was formed by mixing the Matrigel (Growth factor-reduced phenol red-free Matrigel, #356231, BD Biosciences) with transfected cells diluted in PBS in a 1:1 ratio. Using forceps, a nylon mesh was gently placed on the surface of the CAM and 50 µL of the Matrigel-cell mixture was pipetted directly onto the meshes, with two on-plants of the same treatment placed on each egg. The eggs were then returned to the incubator until EDD 11 where they were sacrificed by placing on ice for 30 min. Surgical scissors and forceps were used to gently lift the CAM and cut it from the rest of the egg. The CAM was then rinsed off in PBS, turned over to view the underside of the on-plant, and imaged immediately using the Leica surgical microscope and camera. Each experiment was carried out in duplicate, with results combined to ensure enough data points for a reliable statistical analysis [60].

### 2.13. Quantification of Angiogenesis

Any chicks which had perished during the course of the experiment were excluded from the analysis. With the analyst blinded to the treatment used for each on-plant, angiogenesis as a result was scored by determining the vascular index of blood vessels which grew towards the on-plant, similarly, to as described previously [37,62,63]. In the case of the CAM filter disc assay, scores of 0, 1, or 2 were assigned to each blood vessel if the angle at which it approached the on-plant (⌀ 6 mm, A = 28.27 mm^2^) was greater than 45°, and whether it branched within a ring (⌀ 12 mm) encircling each filter disc. Similarly, in the case of the Matrigel-cell experiments, all blood vessels which approached the on-plant (A = 16 mm^2^) at an angle greater than 45° were assigned a score of 0, 1, or 2 based on whether they branched within the focal plane of the on-plant. In the case of both experiments, the total score for each on-plant was then determined by the sum of each of these individual scores.

### 2.14. Statistical Analysis

All statistical analyses were carried out using Excel and GraphPad Prism software. A Student’s *t*-test was used to determine statistical significance between the two groups. A one-way analysis of variance (ANOVA) and Tukey’s post hoc test were applied to determine statistical significance between groups, while a two-way analysis of variance (ANOVA) was applied where two factors were present in an experiment. Data are expressed as mean ± S.E.M. or as a scatter plot ± S.E.M., with data considered statistically significant at * *p* < 0.05; ** *p*  <  0.01; *** *p*  <  0.001 and **** *p*  <  0.0001.

## 3. Results

### 3.1. hVEGF Stable Transfected C2C12 Cells Exhibit Continued G418 Resistance Compared to Non-Transfected Cells

C2C12 muscle cells were transfected with pEGFPN1, pVEGF-EGFPN1, and FGF4/VEGF plasmids. Over the course of four weeks following transfection, G418 antibiotic selection was carried out. Two clones of each pEGFPN1 and pVEGF-EGFPN1 and eleven clones of FGF4/VEGF transfected cell colonies, which showed continued antibiotic resistance were selected and expanded to larger numbers. Based on the brightness of their GFP activity, pEGFPN1 and pVEGF-EGFPN1 clones were referred to as low and high expression pEGFPN1 and p-VEGF-EGFPN1 (Figure S3A). Once established, these clones were monitored closely as it was imperative that antibiotic resistance continued to be observed to ensure successful stable transfection.

### 3.2. hVEGF Stable Transfected Cells Express Significantly Increased hVEGF-A Protein Compared to GFP Transfected and Non-Transfected C2C12 Cells

**Figure 1:**
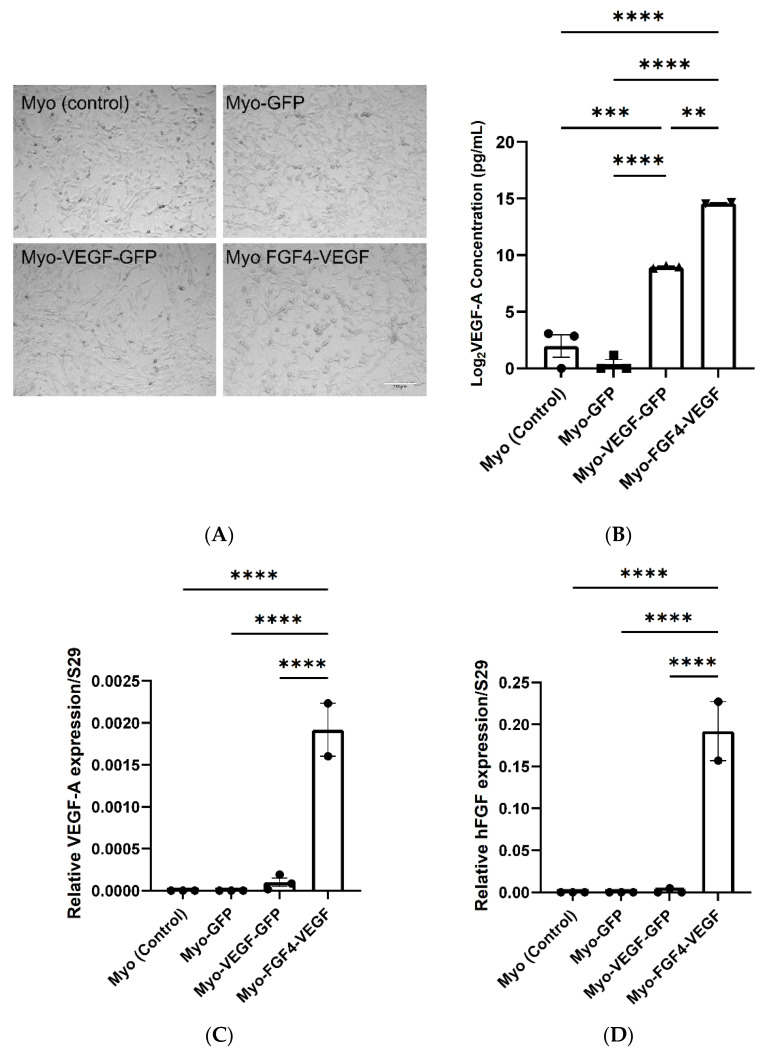
Analysis of VEGF-A and FGF4 expression in transfected C2C12 myoblast cells. (**A**) Stably transfected cell lines; Myo (control), Myo-GFP, Myo-VEGF-GFP, and Myo-FGF4-VEGF. Taken at 10x magnification. Scale bar = 200 μm (**B**) VEGF-A secretion into cell media (DMEM). Supernatant media was removed from confluent cells after 72 h. A one-way ANOVA with Tukey’s post hoc test indicates a significant increase in VEGF-A production in Myo-VEGF-GFP and Myo-FGF4-VEGF cells compared to Myo-GFP and Myo-control cells. Data was Log2 transformed and is presented as mean ± SEM with *n* = 2–3. (**C**) RNA was extracted from confluent Myo-control, Myo-GFP, Myo-VEGF-GFP, and Myo-FGF4-VEGF cell lines. RNA reverse transcribed into cDNA was analysed using qPCR. Results were normalised relative to the expression of the ribosomal S29 housekeeping gene. No VEGF-A expression was observed in Myo (control) or Myo-GFP cells. A modest upregulation in VEGF-A expression is seen in Myo-VEGF-GFP cells, while a significant upregulation is seen in Myo-FGF4-VEGF cells. (**D**) No *hFGF4* expression is observed in Myo (control), Myo-GFP, and Myo-VEGF-GFP cells, while a significant upregulation in FGF4 expression is seen in Myo-FGF4-VEGF cells. Data presented as mean ± SEM; *n* = 2–3. A similar pattern is observed in the expression of h-VEGF-A protein and gene expression in each cell line. A significant increase in VEGF-A protein and gene expression is seen in Myo-FGF4-VEGF cells compared to Myo (control) and Myo-GFP cell lines, while a more modest increase is seen in Myo-VEGF cell lines. Data was analysed by one-way ANOVA and Tukey’s multiple comparison post-hoc test; ** *p* < 0.05, *** *p* < 0.01, **** *p* < 0.001.

Two angiogenic and two control cell lines were established and named Myo-VEGF-GFP, Myo-FGF4/VEGF, Myo (control), and Myo-GFP (Figure 1A). A human VEGF-A ELISA was used to validate increased *hVEGF-A* protein expression in stably transfected cells, and to screen cells to identify those with the most potency. To achieve this, the level of *hVEGF-A* protein released from the stably transfected cell lines into the cell media was measured. Non-transfected C2C12 and pEGFPN1 transfected cells served as controls, where no *hVEGF-A* expression is expected. High expression pVEGF-EGFN1 transfected cell lines showed a high *hVEGF-A* secretion compared to pEGFPN1 and non-transfected C2C12 cell lines (*p* < 0.0001) (Figure S3B). While clones 3, 5, and 11 of FGF4/VEGF transfected cell lines also showed a high level of *hVEGF-A* protein expression (*p* < 0.0001) (Figure S3C). Based on this *hVEGF-A* secretion, specific stable transfected cell lines were selected and established for each plasmid to be used for further investigation into their angiogenic potential: pEGFPN1 low expression named Myo-GFP, pVEGF-EGFN1 high expression named Myo-VEGF-GFP and FGF4/VEGF clone 3 named Myo-FGF4/VEGF. Myo-FGF4/VEGF cell line exhibited a potent VEGF-A secretion, secreting a 5000–50,000-fold surplus in VEGF-A production compared to Myo (control) and Myo-GFP cell lines, and an almost 50-fold increase compared to Myo-VEGF-GFP cell lines (Figure 1B).

### 3.3. hVEGF-A and hFGF4 mRNA Expression in C2C12 Cells

Additionally, we performed qPCR to analyse *hVEGF* and *hFGF4* expression in the Myo (control), Myo-GFP, Myo-VEGF-GFP, and Myo-FGF4-VEGF cell lines to confirm altered gene expression. qPCR indicated a significant upregulation of *hVEGF* expression in Myo-VEGF-GFP and Myo-FGF4-VEGF cell lines compared to non-transfected and Myo-GFP cell lines which resulted in undetermined products. A modest increase of *hVEGF* was seen in the Myo-VEGF-GFP cell line. Meanwhile, a much more apparent increase was seen with the Myo-FGF4-VEGF cell line compared to Myo (control) and Myo-GFP cell lines (*p* < 0.0001) (Figure 1C). *hFGF4* expression in the Myo-FGF4-VEGF cell line was also highly detected compared to Myo (control), Myo-GFP, and Myo-VEGF-GFP (*p* < 0.0001) cells (Figure 1D), confirming the dual expression of both *hFGF4* and *hVEGF-A* in this cell line. These data further confirmed the initial ELISA results, supporting the findings that stably transfected cell lines express significant levels of *hVEGF-A* alone, or in combination with *hFGF4*, respectively.

### 3.4. Myo-VEGF-GFP and Myo-FGF4-VEGF Conditioned Media Increase Proliferation, Migration, and Tube Formation in Endothelial Cells

Following confirmation of *hVEGF-A* expression and secretion by Myo-VEGF-GFP and Myo-FGF4-VEGF cells, our next aim was to investigate the angiogenic potential of the conditioned media from these different cell lines. Both *hVEGF-A* and hFGF play important roles in mediating endothelial cell growth and promoting angiogenesis, as a result, endothelial cell angiogenic assays were employed. This involved monitoring the behaviour of C166 endothelial mouse yolk-sac cells, following treatment with conditioned DMEM taken from each cell line.

**Figure 2:**
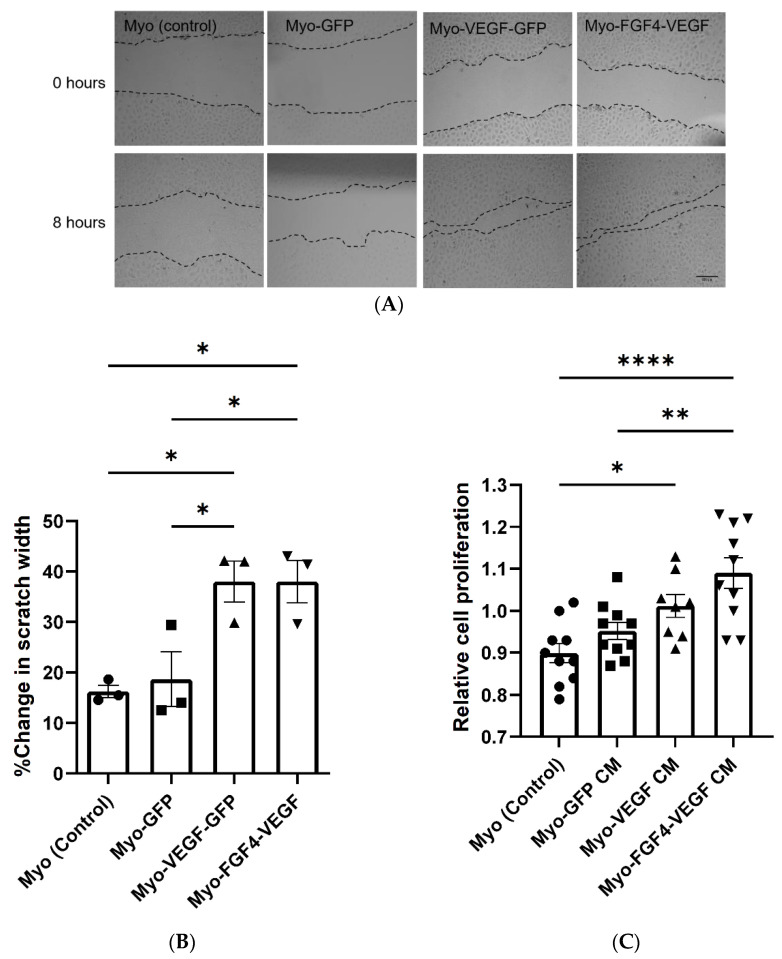
Conditioned media from stably transfected myoblasts stimulates endothelial cell migration. The behaviour of C166 endothelial cells was observed following treatment with conditioned media (CM) taken from stably transfected cell lines. (**A**) Representative images of in vitro scratch assay to analyse C166 endothelial cell migration following treatment with stable cell CM for 8 h. Images taken at 10X magnification. Dotted lines indicate the margins of each scratch. Scale bar = 200 μm. (**B**) Cell migration was evaluated by measuring the width of scratch at both time points using ImageJ Software. Results are expressed as the percentage of scratch closure following each treatment. A significant increase in migration of endothelial cells following Myo-VEGF-GFP and Myo-FGF4-VEGF CM treatment compared to CM from Myo-GFP and Myo (control) cells; *n* = 3. (**C**) Conditioned media from stably transfected myoblasts stimulates endothelial cell proliferation. A mitochondrial metabolic (MTT) activity assay was carried out to determine C166 cell proliferation following treatment for 72 h with CM taken from confluent stable transfected cells. A significant increase in cell proliferation following treatment with Myo-VEGF-GFP CM compared to non-transfected CM and Myo-FGF4-VEGF CM compared to both Myo-GFP and Myo (control) CM. *n* = 8–10. Data were analysed by one-way ANOVA with Tukey’s post hoc test. All data presented as mean ± SEM; * *p* < 0.05; ** *p* < 0.01; **** *p* < 0.0001.

In a scratch assay, the migration of endothelial cells can be observed following the formation of a scratch in the cell monolayer, with increased or decreased rates of migration seen because of various treatments. In Figure 2A, visually this migration of cells into the scratch formed can be seen over the 8 h of observation, with increased migration seen as a result of conditioned media taken from stably transfected cell lines. Measurement of this scratch before and 8 h after treatment, indicated a significant increase in the % change in scratch width as a result of Myo-VEGF-GFP and Myo-FGF4-VEGF conditioned media compared to media from Myo (control) or Myo-GFP cell lines, with *p* < 0.05 (Figure 2B). Complimentary to this, as measured using an MTT assay, conditioned media taken from Myo-FGF4-VEGF cell lines induces a significant increase in the proliferation of endothelial cells compared to media from Myo (control) (*p* < 0.0001) or Myo-GFP (*p* < 0.0051) cell lines. Myo-VEGF-GFP cell conditioned media elicited a significant increase in cell proliferation compared to non-transfected cell conditioned media (*p* < 0.05) (Figure 2C). Interestingly, no significant increase in proliferation or migration was observed as a result of Myo-FGF4-VEGF conditioned media compared to Myo-VEGF-GFP, despite the stark difference in VEGF-A protein levels.

**Figure 3:**
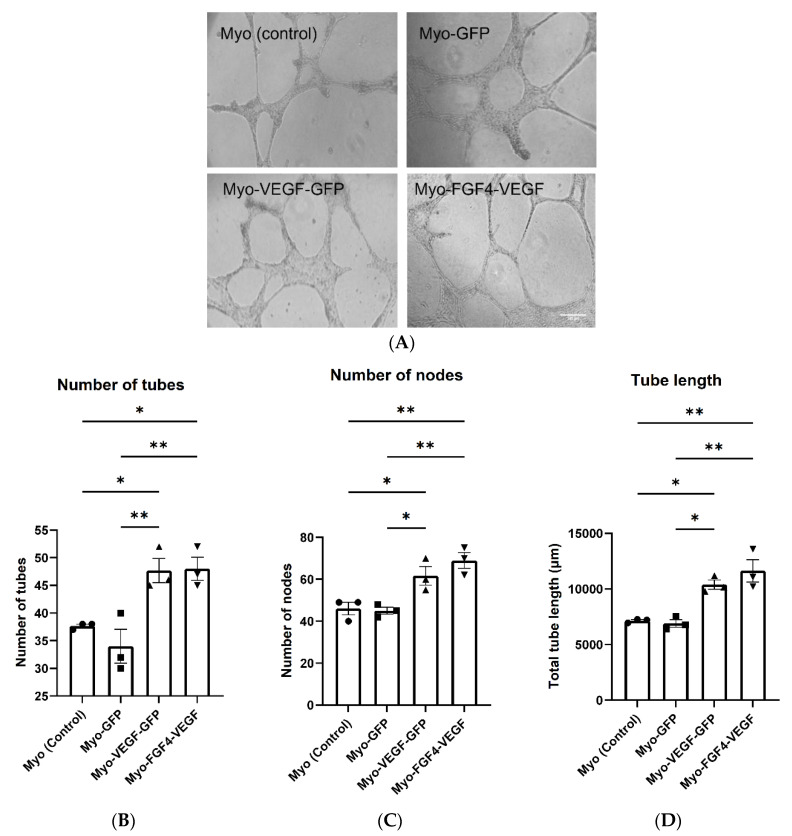
Conditioned media from stably transfected myoblasts stimulates endothelial cell tube formation. (**A**) Representative images of Endothelial cell tube formation assay as a result of CM taken from confluent stable transfected cells. C166 Cells were suspended in CM on Matrigel and observed for 18 h until tube-like structures formed. Images were taken at 10X magnification. Scale bar = 200 μm. Four images were obtained at non-overlapping locations with (**B**) number of tubes (**C**) number of nodes and (**D**) total tube length quantified using Image J software. A significant increase in tube length and the number of tubes and nodes quantified following Myo-VEGF-GFP and Myo-FGF4-VEGF CM treatment compared to CM from GFP and Myo (control) cells. *n* = 3; Data was analysed by one-way ANOVA with Tukey’s post hoc test. All data presented as mean ± SEM. * *p* < 0.05; ** *p* < 0.01.

When seeded on a basement membrane matrix with the correct angiogenic factors present, endothelial cells can begin to form a well-defined tubular network. Following the application of C166 mouse endothelial cells suspended in conditioned media taken from stable cell lines applied to a Matrigel base in 96-well plates, the formation of this well-defined network was observed (Figure 3A). Four images were obtained at non-overlapping locations following treatment with conditioned media from each cell line. Quantification of these images indicated a significant increase in the number of tubes, nodes and total tube length following treatment with Myo-VEGF-GFP and Myo-FGF4-VEGF conditioned media compared to media from Myo (control) and Myo-GFP cells, with *p* < 0.05 (Figure 3B–D). However, despite the potent VEGF-A secretion of the Myo-FGF4-VEGF cell line compared to Myo-VEGF-GFP, little difference is seen in the parameters of the number of tubes, nodes, and overall tube length.

These results indicated that through migration, proliferation, and tube formation assays, conditioned media taken from these *hVEGF-A* and *hFGF4* secreting cells elicited angiogenic-like behaviours in endothelial cells, indicating the pro-angiogenic potential of these cell lines.

### 3.5. Conditioned Media from Myo-VEGF-GFP and Myo-FGF4-VEGF Cells Leads to Increased Angiogenesis in the CAM

**Figure 4:**
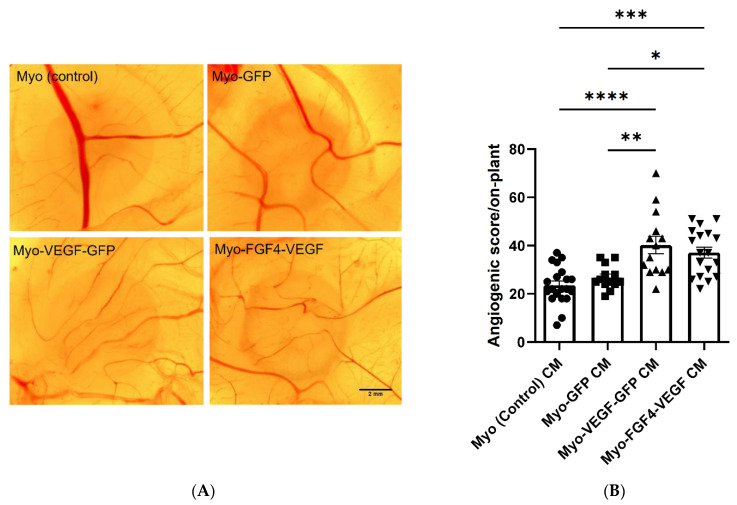
Angiogenic response of CAM following treatment with CM from stable transfected C2C12 cells. (**A**) Representative images of CAM following 72-h treatment with filter paper discs saturated with CM taken from confluent Myo (control), Myo-GFP, Myo-VEGF-GFP, and Myo-FGF4-VEGF cells. Images were taken at 16X magnification. Scale bar = 2 mm (**B**) Angiogenic score of filter discs. A one-way ANOVA with Tukey’s post hoc test indicates a significant increase in the angiogenic score as a result of Myo-VEGF-GFP or Myo-FGF4-VEGF cells compared to Myo-GFP or Myo (control) cells. Data presented as mean ± SEM with *n* = 13–20. * *p* < 0.05; ** *p* < 0.01; *** *p* < 0.001; **** *p* < 0.0001.

The CAM filter disc assay was employed to further validate our findings of the angiogenic potential of *hVEGF-A* producing stably transfected cell lines. Conditioned media from each of the confluent stable transfected cell lines were applied to the CAM assay, delivered via a saturated filter paper disc, with the angiogenic response observed. An increased level of angiogenesis was observed with conditioned media from *hVEGF-A* secreting cell lines compared to non-transfected or control transfected cell lines (Figure 4A). Following quantification of angiogenic scores of each treatment group, Myo-VEGF-GFP and Myo-FGF4-VEGF cell lines elicited a 1.5-fold increase in the angiogenic score compared to Myo (control) and Myo-GFP cell lines (Figure 4B). These data further demonstrate the pro-angiogenic potential of conditioned media taken from these stably transfected cells.

### 3.6. Myo-VEGF-GFP and Myo-FGF4-VEGF Cells Directly Elicit Increased Angiogenesis in the CAM

Following confirmation that conditioned media from these stably transfected cells elicited pro-angiogenic effects in both endothelial cell angiogenic assays and the CAM filter disc assay, the focus of this study then moved to the investigation of the angiogenic response of the gene-modified cells directly. To examine this, Matrigel-cell mixtures were applied to the CAM. To begin, a dose-dependent curve using the CAM assay was carried out, with increasing quantities of Myo-VEGF-GFP and Myo-FGF4-VEGF cell lines respectively applied to the CAM for 72 h to establish the optimum cell number required (Figure S4A,B). For both Myo-VEGF-GFP and Myo-FGF4-VEGF cells, 1.5 × 10^6^ cells/on-plant was seen as the optimum concentration of cells to elicit a significant angiogenic effect, with *p* < 0.05 (Figure S4C). However, in the case of Myo-FGF4-VEGF, increased concentrations of over 1.5 × 10^6^ cells appear to have elicited a negative response on angiogenesis in the CAM, with a significant decline in angiogenic score observed (Figure S4D). Based on this, the angiogenic effects of on-plants consisting of 1.5 × 10^6^ cells/on-plant for Myo (control), Myo-GFP Myo-VEGF-GFP, and Myo-FGF4-VEGF were investigated through an application on developmental day 8 (EDD8) for 72 h.

**Figure 5:**
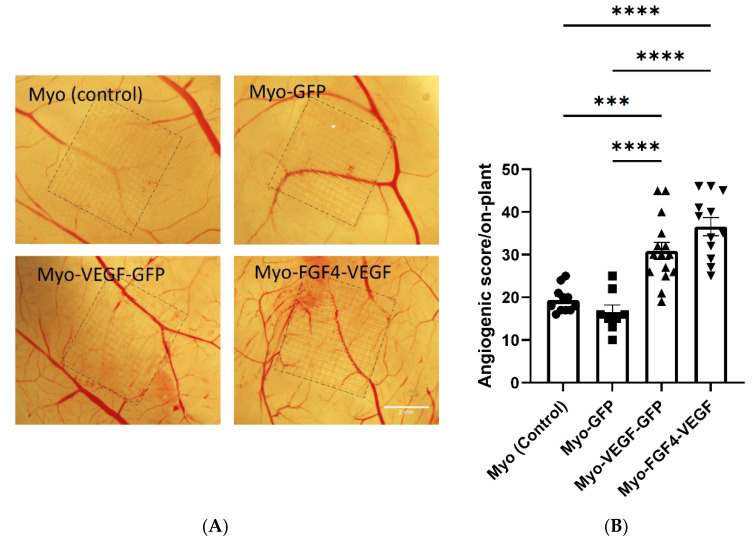
Angiogenic response following Matrigel and stably transfected cell on-plants using CAM assay. (**A**) Representative images of the angiogenic response of Matrigel-cell on-plants containing 1.5 × 10^6^ stable transfected cells were applied to the CAM assay on EDD8 for 72 h. Images were taken at 25X magnification. Scale bar= 2 mm. (**B**) Angiogenic score of Matrigel-cell on-plants. A one-way ANOVA with Tukey’s post hoc test indicates there is a significant increase in angiogenic score in Myo-VEGF-GFP and Myo-FGF4-VEGF cells compared to Myo-GFP and Myo (control) cells. Data presented as mean ± SEM with *n* = 8–15. *** *p* < 0.001; **** *p* < 0.0001.

Following the application of Matrigel encapsulated cell on-plants, visually increased angiogenesis was seen as a result of both Myo-VEGF-GFP and Myo-FGF4-VEGF cell lines compared to Myo (control) and Myo-GFP encapsulated on-plants respectively (Figure 5A). An angiogenic score was calculated for each on-plant based on the number, branching, and angle of blood vessels approaching a square drawn around the on-plant. Application of Myo-VEGF-GFP cells to the CAM assay resulted in a significant increase in angiogenic response compared to Myo (control) (*p* < 0.001) and Myo-GFP cells (*p* < 0.0001). Meanwhile, Myo-FGF4-VEGF cells elicited a similar response, resulting in a significant increase in angiogenic observed compared to on-plants containing Myo (control) and Myo-GFP cells, with *p* < 0.001 (Figure 5B).

## 4. Discussion

VEGF is paramount in vascular development and is the single most important regulator of blood vessel formation [14,64]. Angiogenesis is a complex, coordinated process involving many growth factors whereby new blood vessels form from pre-existing vessels [3]. VEGF-A has a vital role in this process with the ability to regulate the migration, proliferation, and endothelial tube formation, and has been the focus for targeted pro-angiogenic therapy [14,15,16,65]. Both VEGF and FGF and their receptors have been identified as paramount in tumour angiogenesis and serve as the main target in potential anti-angiogenic cancer therapies [66,67]. Tumours require adequate blood supply for survival, with the “angiogenic switch” essential in developing this vascularisation, by triggering the release of growth factors such as FGF and VEGF [68].

We conducted a number of studies in order to establish stable *hVEGF-A* secreting C2C12 mouse myoblast cell lines, and quantify the angiogenic potential of these cells, which may prove beneficial in the amelioration of muscle ischaemia associated with diseases including CLI, PAD, diabetes, cachexia, COPD and muscular dystrophy [4,6].

In the present study, we successfully established two stable muscle cell lines including expression of human VEGF-A protein, Myo-VEGF-GFP as a GFP tagged fusion protein, and Myo-FGF4-VEGF as cells dually expressing human FGF4 and *hVEGF*. Conditioned media taken from the *hVEGF-A* secreting cell lines proved efficacious in the induction of angiogenesis in the CAM angiogenic assay, while also resulting in pro-angiogenic behaviours in murine endothelial cell assays of angiogenesis. Finally, following confirmation of the pro-angiogenic benefits as a result of conditioned media, we then demonstrated how these cells provoked a potent angiogenic response in the CAM angiogenic assay.

The efficacy of myoblast-mediated VEGF gene delivery to mouse and rat muscle has been investigated in the past, with varying levels of success. Despite the potent angiogenic potential of VEGF, studies have indicated that the optimum dosage for VEGF is within a narrow limit, with overproduction leading to the formation of disorganized, malformed vessels with irregularly sized lumens and weakened wall permeability [20,69,70,71]. Angiogenesis is a complex process requiring the delicate balance of several growth factors. Co-delivery of VEGF-A with another growth factor, such as fibroblast growth factor (FGF) or placental derived growth factor (PDGF) may counteract these aforementioned issues, allowing for the development of healthy mature blood vessels [7,18,25,26]. FGF has been demonstrated to have a synergistic effect with VEGF-A [22,72], it has been proven to increase endothelial cell mitogenic activity and induce therapeutic angiogenesis in the rabbit hind-limb ischemia model [73]. Therefore, in this study, we chose to explore the angiogenic efficacy of cell lines expressing *hVEGF-A* alone and in combination with *hFGF4*.

Previous studies have generated *hVEGF-A* and *hFGF4*/*VEGFA* overexpressing C2C12 mouse myoblast cells, both as a result of stable and transient transfections [18,26]. In the present study, the concentrations of *hVEGF-A* secretions in the dual expression cell line are much higher than in previous reports, confirmed by both ELISA and qPCR [18,25,26,74,75]. Following several weeks of G418 antibiotic selection, measurement by both ELISA and qPCR, reported elevated *hVEGF-A* protein secretion and mRNA from both cell lines, while qPCR alone confirmed the dual expression of *FGF4* and *VEGF* in the Myo-FGF4-VEGF cell line. Myo-FGF4-VEGF cells secreted a significantly higher amount of *hVEGF-A* protein compared to non-transfected and Myo-GFP cell lines, while in comparison to the Myo-VEGF-GFP cell line, Myo-FGF4-VEGF cells exhibited an almost 50-fold increase in *hVEGF-A* protein secretion.

Research to date has examined the expression and therapeutic benefits of both VEGF and FGF secretions separately [76]. However, the simultaneous co-delivery of both growth factors in a single cell line remains relatively unexplored [77]. The dual FGF4-ires-VEGF plasmid is a bicistronic expression vector, which allows the simultaneous translation of two genes from a single mRNA transcript. In this plasmid, *hVEGF* falls under IRES-dependent second gene expression, as a result, expression of *hVEGF* would be expected to be lower compared to the single VEGF expression plasmid [78]. This can be seen in the qPCR results, wherein the Myo-FGF4-VEGF cell line, the *hFGF4* gene transcript level is higher than the *hVEGF-A* expression.

The dual FGF4/VEGF plasmid used in this study has been successfully transfected into myoblast cells in previous reports [18,25,74]. However, the level of *hVEGF-A* protein secretion in these studies was observed to be much less potent. In previously published reports, transient transfection with this dual FGF4/VEGF plasmid reported *hVEGF-A* secretions of approximately 6000 pg/mL in human primary myoblasts [26] and 2800 pg/mL in C2C12 cells [18]. However, in the latter study, following four weeks of isolation and selection of stably transfected clones, this concentration was almost 25-fold [18]. An AAV gene vector of this dual FGF4/VEGF plasmid has also been used to transduce cells. In Jazwa et al., Hela cells transduced with AAV-FGF4-ires-VEGF reported *hVEGF* secretions of around 5500 pg/mL. Additionally, this was used to ameliorate wound healing and post-ischemic blood flow recovery in the hindlimb ischemic mouse model [25,74].

With the aim of therapeutic angiogenesis, VEGF-165 alone has been transfected into several different cell types including fibroblasts [75], endothelial cells [79], rat myoblasts, and various stem cells [80,81]. Herein, ELISA data reports elevated levels of *hVEGF-A* protein secreted from Myo-VEGF-GFP cells compared to Myo (control) or Myo-GFP transfected cell lines. However, this expression is not as potent as concentrations seen in the Myo-FGF4-VEGF cell lines in the present study. Sun et al., through lentiviral transfection, developed a stable VEGF-165 expressing ADSCs which secreted approximately 920 pg/mL of VEGF [81]. Meanwhile, Wang et al. transduced proliferating cells with an adenovirus harbouring the VEGF-165 gene, eliciting VEGF secretions of around 5000 pg/mL, 14 days post-transduction [79]. This reinforces the variations which can occur in transfections/transductions, while also highlighting the importance of choosing the optimum conditions and delivery methods for successful transfection.

Following on from the successful confirmation of *hVEGF* and *hFGF4*/VEGF protein expression from Myo-VEGF-GFP and Myo-FGF4-VEGF cell lines, we moved our focus to proving the pro-angiogenic effects these cells could elicit. Previously, various cells have been successfully modified or transfected to overexpress VEGF [79,80,82], FGF2 [77] or both [25,74]. However, no research to date has explored the angiogenic effect of *hVEGF* or hFGF4-VEGF expressing C2C12 cells using the chick CAM assay. In the current investigation, we chose to employ both in vitro murine endothelial cell angiogenic assays, and the in vivo chick CAM assay.

VEGF-A and FGF have both proven to elicit pro-angiogenic behaviours in endothelial cells in vitro [83,84]. In the present study, conditioned media taken from Myo-VEGF-GFP and Myo-FGF4-VEGF cell lines, increased murine endothelial cell migration, proliferation, and tube formation. However, here we can assess the potency of *hVEGF* protein secretion in affecting the angiogenic response in vitro. The lower *hVEGF-A* secretion by the Myo-VEGF-GFP cell line is reflected. Consequently, a similar angiogenic response is not as clearly apparent, with Myo-VEGF-GFP conditioned media failing to induce a significant increase in cell proliferation compared to Myo-GFP conditioned media.

The potent angiogenic ability of Myo-VEGF-GFP and Myo-FGF4-VEGF cell conditioned media is again seen in the tube formation assay using endothelial cells seeded on a basement media matrix. In vivo, VEGF triggers the release of proteolytic enzymes which degrade the surrounding basement membrane, allowing endothelial sprouts to invade the extracellular matrix leading to tube formation. Following this, endothelial cells migrate, forming tube-like structures which fuse to form an organised and functional blood vessel network [65]. Conditioned media from both VEGF-A secreting cell lines mimicked this process in vitro, leading to a significant increase in the number of tubes, nodes, and overall length of tubes observed.

Meanwhile, only a modest difference in endothelial cell migration, proliferation, and tube formation is seen in the comparison of Myo-VEGF-GFP and Myo-FGF4-VEGF conditioned media, where despite the increased potency of VEGF-A secretion, little difference in these parameters is observed. This reinforces the delicate VEGF dose-responsive effects aforementioned, where overexpression of VEGF can lead to negative effects [20,85].

While in vitro assays such as the endothelial cell assays can elucidate pro-angiogenic potency, in this study, it was important to examine the efficacy of these cell secretions on the in vivo model of angiogenesis. We chose to use the CAM assay to investigate this pro-angiogenic potential. The CAM assay is a highly versatile, cost-effective model of angiogenesis with a vast vascular network of capillaries, veins, and arteries which can be easily manipulated and observed for experimental study [37,39,40].

A strong and potent angiogenic effect is seen in this study as a result of conditioned media taken from the stable transfected Myo-VEGF-GFP and Myo-FGF4-VEGF cell lines. This is consistent with other studies where conditioned media from osteoblast-like cells which secreted elevated levels of VEGF-A also induced a positive angiogenic response in the CAM assay [86].

The present findings are promising, confirming that Myo-VEGF-GFP and Myo-FGF4-VEGF cell lines secrete elevated levels of *hVEGF* and have the potential to induce angiogenesis in vivo. Naturally, the next phase would be to examine the applicability and efficacy both these cell lines could have in a higher in vivo mouse model of disease. Myoblast transplantation is a promising area of therapeutics [87]; however, it can encounter several biological setbacks in their survival and propagation [18,88]. Therefore, in order to confirm the continued biological activity of these cell lines, the application of the cells directly was carried out, again using the CAM assay as an in vivo model of angiogenesis.

Application of Myo-VEGF-GFP and Myo-FGF4-VEGF cells encapsulated in Matrigel onto the CAM assay resulted in a significant increase in the angiogenic score, similar to the effects seen with cell conditioned media. In this study, the correct dosage of cell number applied was paramount to the most efficient angiogenic response. Higher levels of Myo-FGF4-VEGF cells per on-plant resulted in a reduction in total angiogenic score with a bell-shaped dose-response observed, possibly indicating excessive cell numbers prove to be harmful in the angiogenic potential. This reinforces the potentially deleterious effects of excessive *hVEGF* protein production, as observed in other published studies [20,69,70]. Following application of an optimum quantity of cells per on-plant, almost a 2-fold increase in angiogenesis can be observed as a result of Myo-FGF4-VEGF and Myo-VEGF-GFP cell lines compared to Myo (control) and Myo-GFP cell lines, indicating the potent angiogenic effect of these cells is transferable from in vitro endothelial cell assays to in vivo CAM experimentation.

In summary, this current study demonstrated the ability to deliver growth factors such as *hVEGF-A* and *hFGF4* from C2C12 mouse myoblast cells into an in vivo angiogenic system. These reveal several important findings which could potentially provide broad implications in the field of therapeutic angiogenesis in muscle diseases with defects in microcirculation such as diabetes and muscular dystrophies. We have successfully established two pro-angiogenic mouse myoblast cell lines which secrete *hVEGF-A* alone or in combination with *hFGF4*, with the latter cell line showing great efficacy. Both of these cell lines have proven to elicit a potent pro-angiogenic effect both in vitro in the murine endothelial cell angiogenic assay and in vivo in the chick CAM model of angiogenesis. Furthermore, the manipulation and application of these cells in higher-level in vivo studies may prove highly beneficial in the amelioration of vascular abnormalities in muscle disease.

## Data Availability

Data is contained within the article and Supplementary Materials.

## Supplementary Materials

The following supporting information can be downloaded at: https://www.mdpi.com/article/10.3390/biomedicines10081781/s1, Figure S1: Kill curve of C2C12 cells at day 1, day 3 and day 7 following treatment with various concentrations of G418 antibiotic. Figure S2: Representation of the well-defined network of tubes formed from endothelial cells following seeding on Matrigel. Figure S3: Stable transfection of C2C12 myoblast cells with p-VEGF-EGFPN1, pEGFPN1 and FGF4/VEGF. Figure S4: Dose-dependent angiogenic response of VEGF-A secreting cells on CAM assay. Table S1. The sequences of forward (F) and reverse (R) primers used in quantitative real-time PCR (RT-qPCR.).
