# Supplementary material for: VEGF-A and FGF4 Engineered C2C12 Myoblasts and Angiogenesis in the Chick Chorioallantoic Membrane
Source: Biomedicines. 2022 Jul 23;10(8):1781. doi: 10.3390/biomedicines10081781 (PMC9330725; doi:10.3390/biomedicines10081781)
Supplement: Supplementary file 1 [file biomedicines-10-01781-s001.zip › biomedicines-1768267-supplementary.pdf]

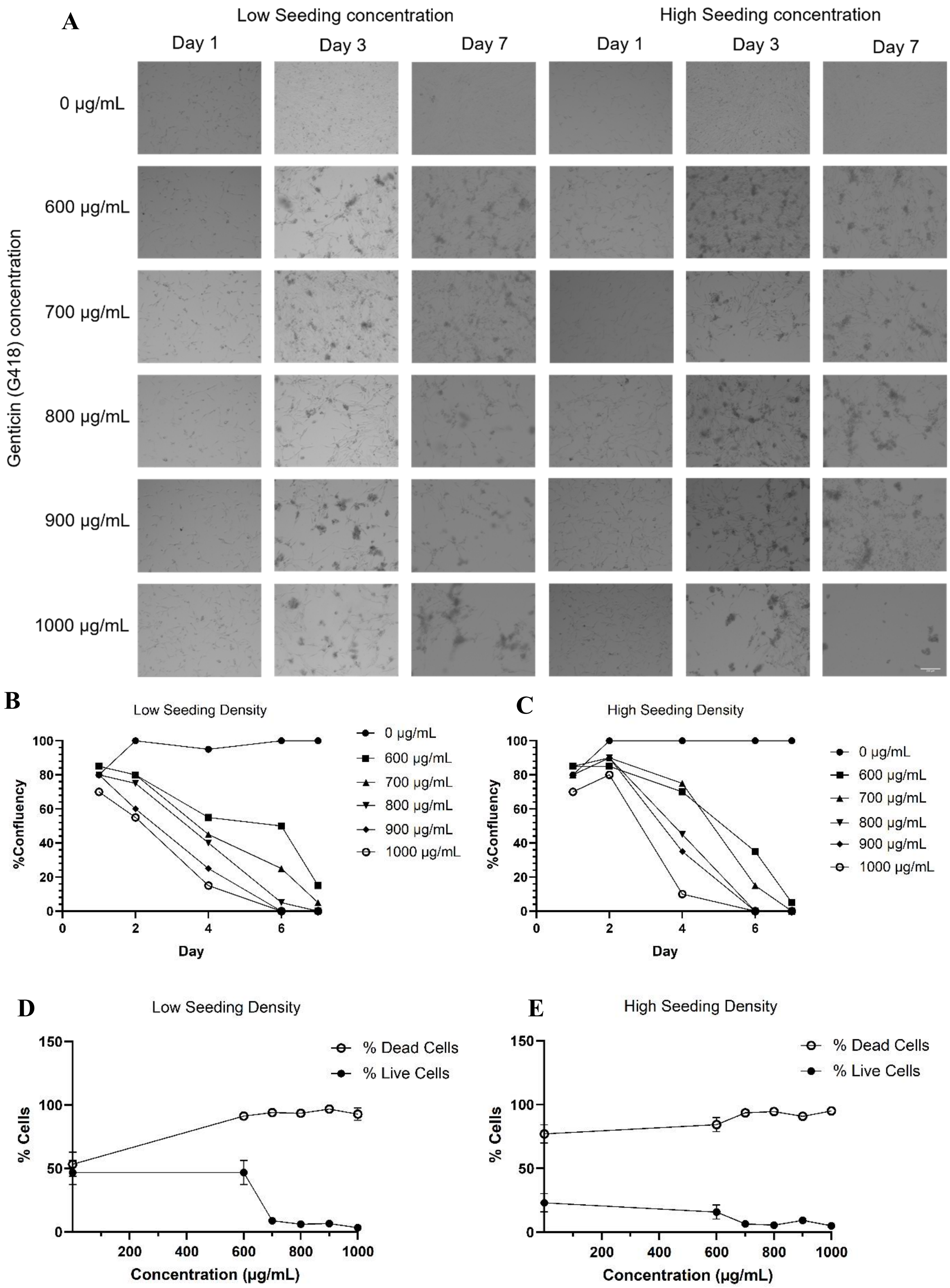

Figure S1. Kill curve of C2C12 cells at day 1, day 3 and day 7 following treatment with various concentrations of G418 antibiotic. (**A**) Representative images of cells seeded both at low ($0.1x10^6$) and high ($0.2x10^6$) concentrations taken at day 1, day 3 and day 7 following treatment with various

concentrations of G418 antibiotic. Visible cell death and toxicity is seen after day 2 at higher antibiotic concentrations. Cells were imaged under brightfield light, captured at 40x magnification, scale bar=200 µm. The confluency of C2C12 cells was visually inspected daily for 7 days following treatment with various concentrations of antibiotic G418. 6-wells plates were seeded at (**B**) low concentration ($0.1x10^6$) and (**C**) high concentration ($0.2x10^6$) with minimum cell viability observed following treatment at a concentration of 800 µg/mL. (**D**,**E**) Percentage cell viability calculated by counting the number of live and dead cells following 7 days of treatment, data is represented as mean percentage of total cells ± SEM with n= 2. Cell viability of less than 10% was observed at a concentration of 1000 µg/mL.

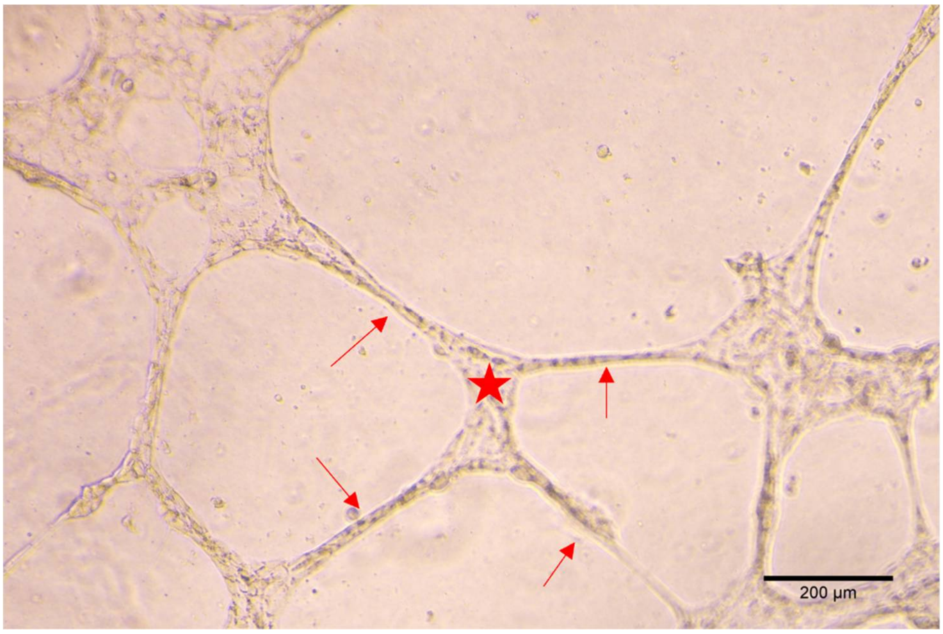

Figure S2. Representation of the well-defined network of tubes formed from endothelial cells following seeding on Matrigel. The red arrows indicate the location of tubes, while the red star indicates a node. Number of tubes, nodes and total tube length were quantified using ImageJ software. Image taken at 10x magnification, scale bar= 200 µm.

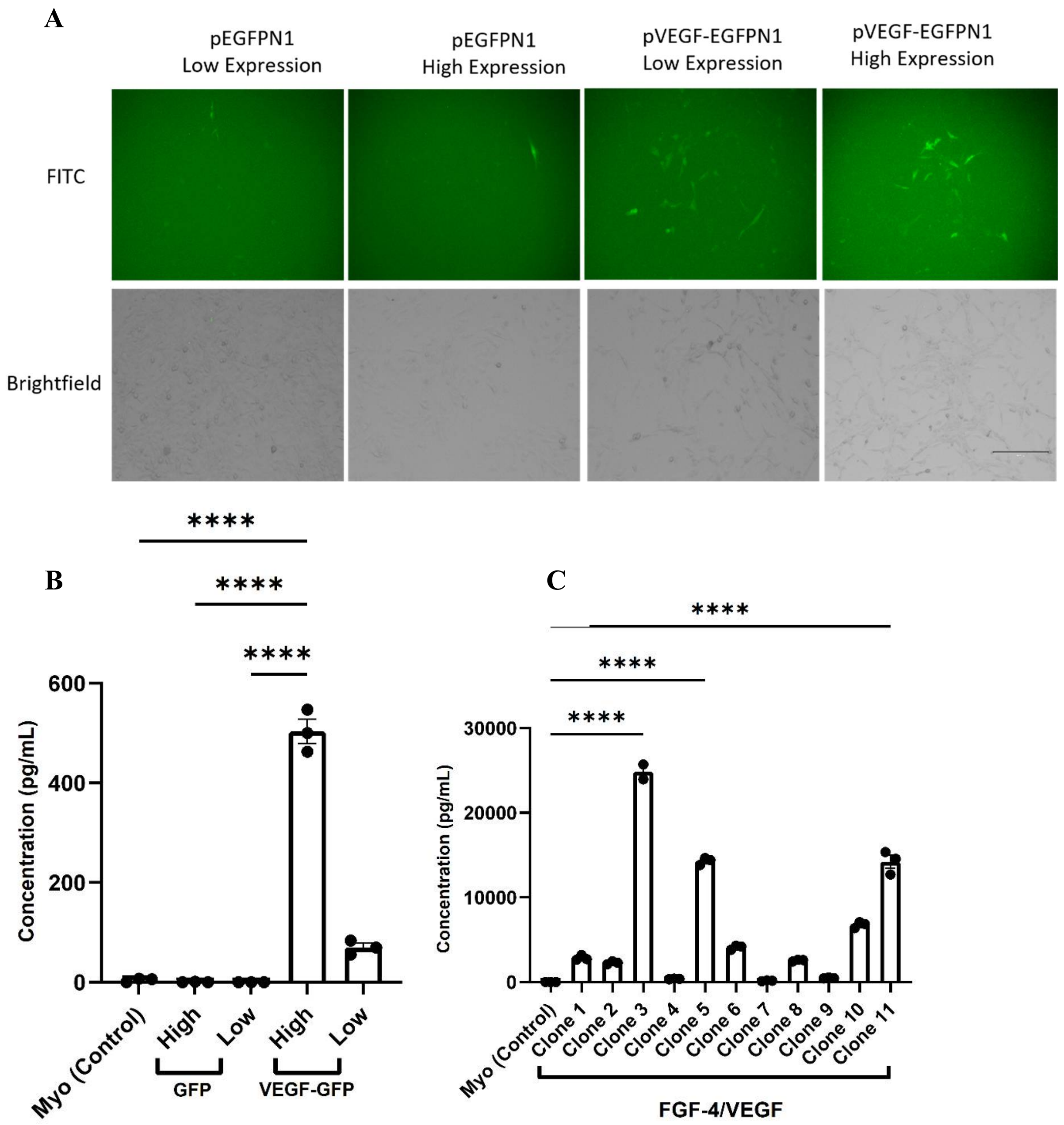

Figure S3. Stable transfection of C2C12 myoblast cells with p-VEGF-EGFPN1, pEGFPN1 and pTR-UF-22-FGF4-ires-VEGF. (**A**) Representative images of selected pEGFPN1 and pVEGF-EGFPN1 stable transfected cell lines following stable transfection and antibiotic selection using 1000 µg/mL G418 for 4 weeks under FITC and Brightfield light. pTR-UF-22-FGF4-ires-VEGF plasmid does not contain a GFP tag, therefore could not be visualised. Images captured under 10x magnification, scale bar=300 µm. (**B**) VEGF-A secretion into cell media (DMEM). Supernatant media was removed from confluent cells after 48 hours. A one-way ANOVA with Tukey's post hoc test indicates a significant increase in VEGF-A production in high expression pVEGF-EGFPN1 cells compared to pEGFPN1 transfected and non-transfected C2C12 cells. (**C**) Similarly, a significant increase in VEGF-A secretion is also seen in pTR-UF-22-FGF4-ires-VEGF transfected clones 3, 5 and 11 compared to Myo (control) cells. Cells which exhibited most potent VEGF-A expression were selected for further analysis of

angiogenic potential and named Myo-GFP, Myo-VEGF-GFP and Myo-FGF4-VEGF. Data presented at mean ± SEM with $n=2-3$; ****$p<0.001$.

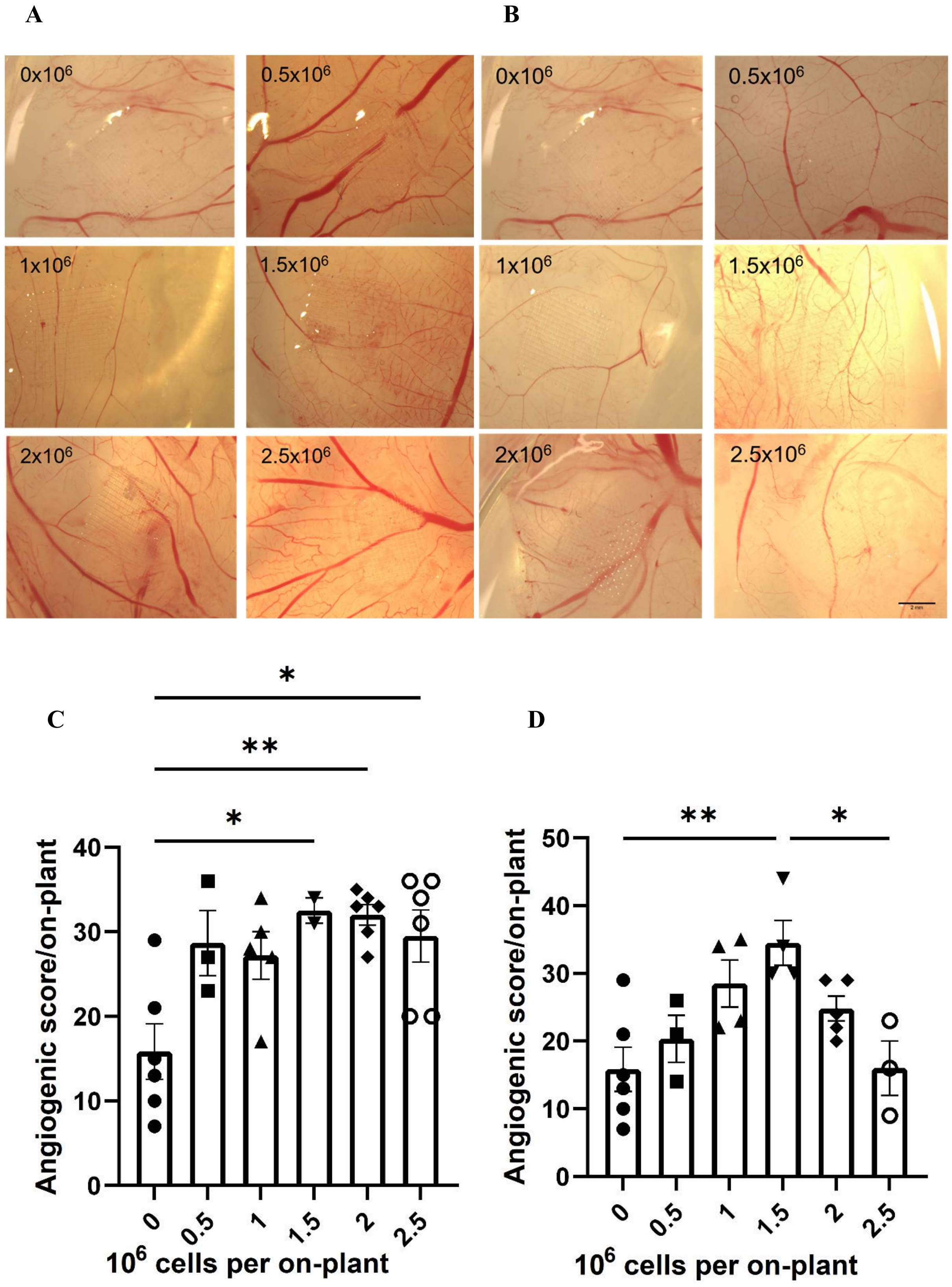

Figure S4. Dose dependent angiogenic response of VEGF-A secreting cells on CAM assay. Representative images of dose dependent angiogenic dose response of on-plants containing increasing amounts of (**A**) Myo-VEGF-GFP and (**B**) Myo-FGF4-VEGF cells applied to the CAM on EDD8 for 72 hours. Images taken at 25x magnification. Scale bar= 2 mm. Dose response of (**C**) Myo-VEGF-GFP

and (**D**) Myo-FGF4-VEGF cell on-plants. A one-way ANOVA with Tukey's post hoc test indicates there is a significant increase in angiogenic score when more than $1.5x10^6$ Myo-VEGF-GFP cells and exactly $1.5x10^6$ Myo-FGF4-VEGF cells are applied compared to Matrigel alone ($p<0.05$). Data presented as dot plot ± SEM with n=2-6. *$p<0.05$; **$p<0.01$.

Table S1. The sequences of forward (F) and reverse (R) primers used in quantitative real-time PCR (RT-qPCR.)

Gene	Gene full name	Sequence
mS29 (Housekeeping gene)	Mouse Ribosomal Protein S29	F: ATGGGTCACCAGCAGCTCTA
		R: GTATTTGCGGATCAGACCGT
hVEGF	Human vascular endothelial growth factor	F: AGATTATGCGGATCAAAC
		R: TTCTTGTCTTGCTCTATC
hFGF	Human fibroblast growth factor	F: CGATGAGTGCACGTTCAAGG
		R: TTCCCATTCTTGCTCAGGGC
